# Trans-cervical thoracic duct embolization for post-surgical left Chylothorax in a patient with multifocal lymphatic malformations

**DOI:** 10.1186/s42155-021-00260-4

**Published:** 2021-10-10

**Authors:** Karan Gulaya, Pouya Entezari, Riad Salem, Ahsun Riaz

**Affiliations:** Department of Radiology, Section of Interventional Radiology, Northwestern Memorial Hospital, 676 N St. Clair, Suite 800, Chicago, IL 60611 USA

**Keywords:** Thoracic duct embolization, Chylothorax, Lymphatic malformations

## Abstract

**Background:**

Mediastinal and abdominal lymphatic malformations may not be diagnosed until adulthood. Radiologic and pathologic diagnosis is often challenging due to the rarity of the lesion. Surgical excision of these lesions may be curative but lymphatic leak is a known complication. Lymphatic duct embolization may then be required to treat the leak.

**Case presentation:**

We describe a patient with post-surgical chylothorax where thoracic duct lymphangiography and embolization was performed by catheterizing the thoracic duct at the venous angle where it drains into the subclavian vein.

**Conclusion:**

Lymphatic duct embolization can be challenging in patients with lymphatic malformations. In these patients, if there is adequate visualization on ultrasound or fluoroscopy, terminal aspect of the thoracic duct can be accessed through the subclavian vein to perform the procedure.

## Introduction

Lymphatic malformations are mature endothelium lined vessels occurring in abnormal clusters that are usually diagnosed in childhood when superficial (Itkin & McCormack, 2016). They are classified as slow flow vascular malformations that usually present as a painless mass with slow growth in the pediatric population. Rarely, lymphatic malformations may occur in the chest or abdomen and escape diagnosis until adulthood (Francavilla et al., 2017). Symptoms are usually related to mass effect or cosmetic deformity. Treatment consists of surgery, percutaneous sclerotherapy, or a combination of the two based on lesion location, size, and symptoms. When lymphatic malformations are not detected until adulthood, they may represent diagnostic challenges given their rarity. Percutaneous biopsy is usually indicated when imaging suggests a mass lesion, although, this is often non-diagnostic and surgery may then be performed. One complication of surgery may be persistent chyle leak and the development of chylothorax, which often requires thoracic duct embolization for resolution (Itkin et al., 2009).

### Technique

**Fig. 1:**
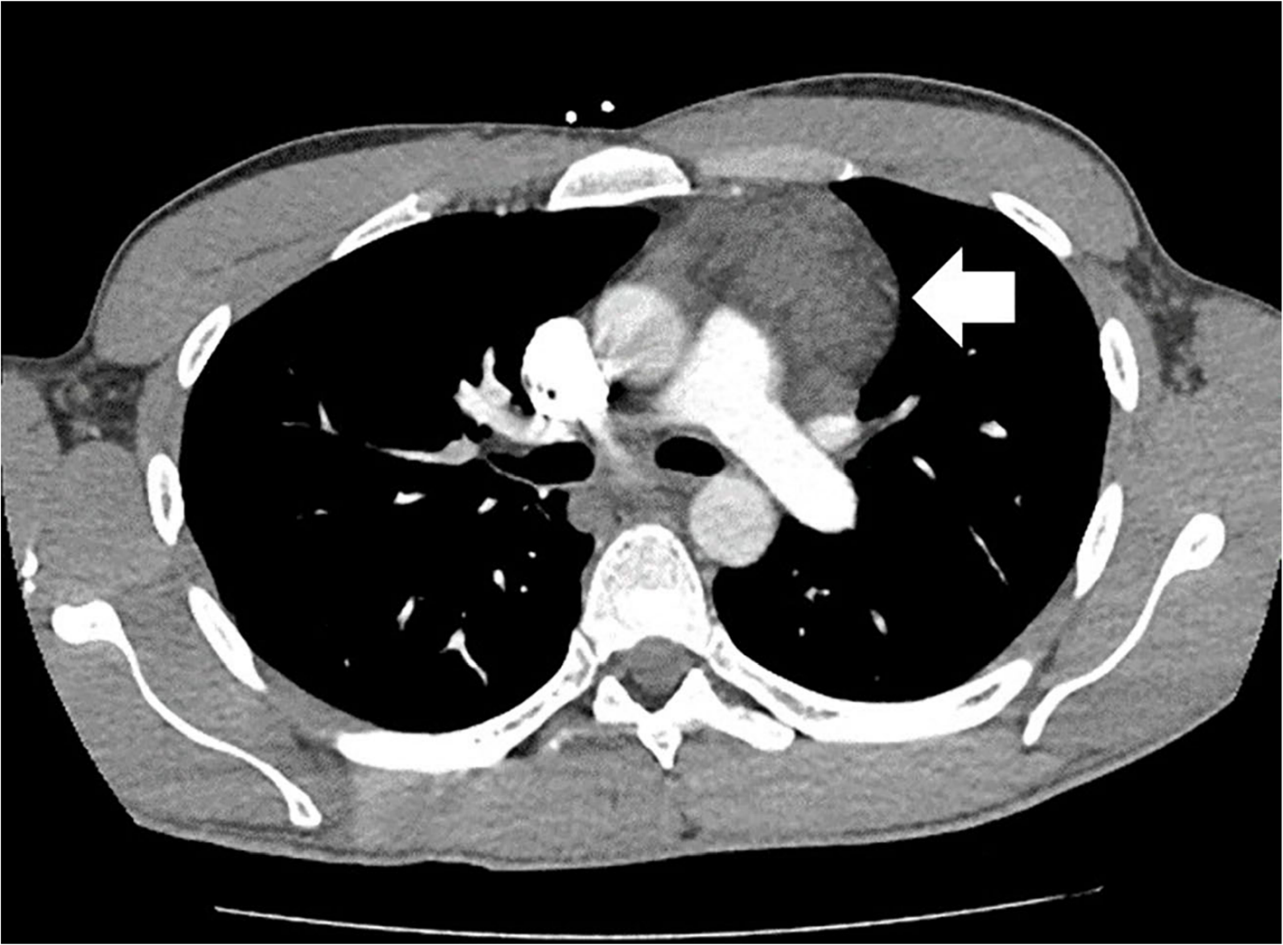
Low density anterior mediastinal mass (white arrow) identified on contrast enhanced CT

A young man presented with a 1-year history of intermittent fatigue, fever, cough, and lower extremity edema. On exam he had palpable fullness in the left supraclavicular region without a discrete mass. He had no inguinal or axillary lymphadenopathy and testicular exam was normal. He had a strong family history of hematologic/immunologic malignancies including non-Hodgkin’s lymphoma, leukemia, and Waldenstrom’s macroglobulinemia. Work-up and imaging revealed hypoalbuminemia and multifocal low-density masses in the retroperitoneum and anterior mediastinum suggestive of widespread lymphadenopathy (Fig. 1). Infectious disease panel was negative except for Cytomegalovirus (CMV) IgG positivity. Serum Lactate dehydrogenase (LDH) and uric acid levels were within normal limits. Lymphoma was the leading differential diagnosis initially and the patient underwent a positron emission tomography/ Computed tomography (PET/CT) followed by left supraclavicular lymph node excisional biopsy. The PET/CT demonstrated no hypermetabolic activity corresponding to the low-density lymphadenopathy in the chest and abdomen. Left supraclavicular lymph node biopsy was insufficient for diagnosis showing fragments of fibro-adipose tissue with patchy dense lymphoplasmacytic infiltrate in a background of fibrosis but no evidence of a monoclonal neoplasm. By this time, he had been evaluated by specialists in hematology/oncology, thoracic surgery, infectious disease, gastroenterology, and general surgery.

The patient was referred back to thoracic surgery for a left video-assisted thoracoscopic surgery (VATS) excision of the anterior mediastinal mass to obtain a larger tissue sample for diagnosis. The procedure was uneventful but he later developed high volume left chylous effusion. He was started on total parenteral nutrition and octreotide. On postoperative day 15, he still had high volume chylothorax draining up to 800 mL daily. Final surgical pathology revealed a benign lymphatic-venous malformation.

**Fig. 2:**
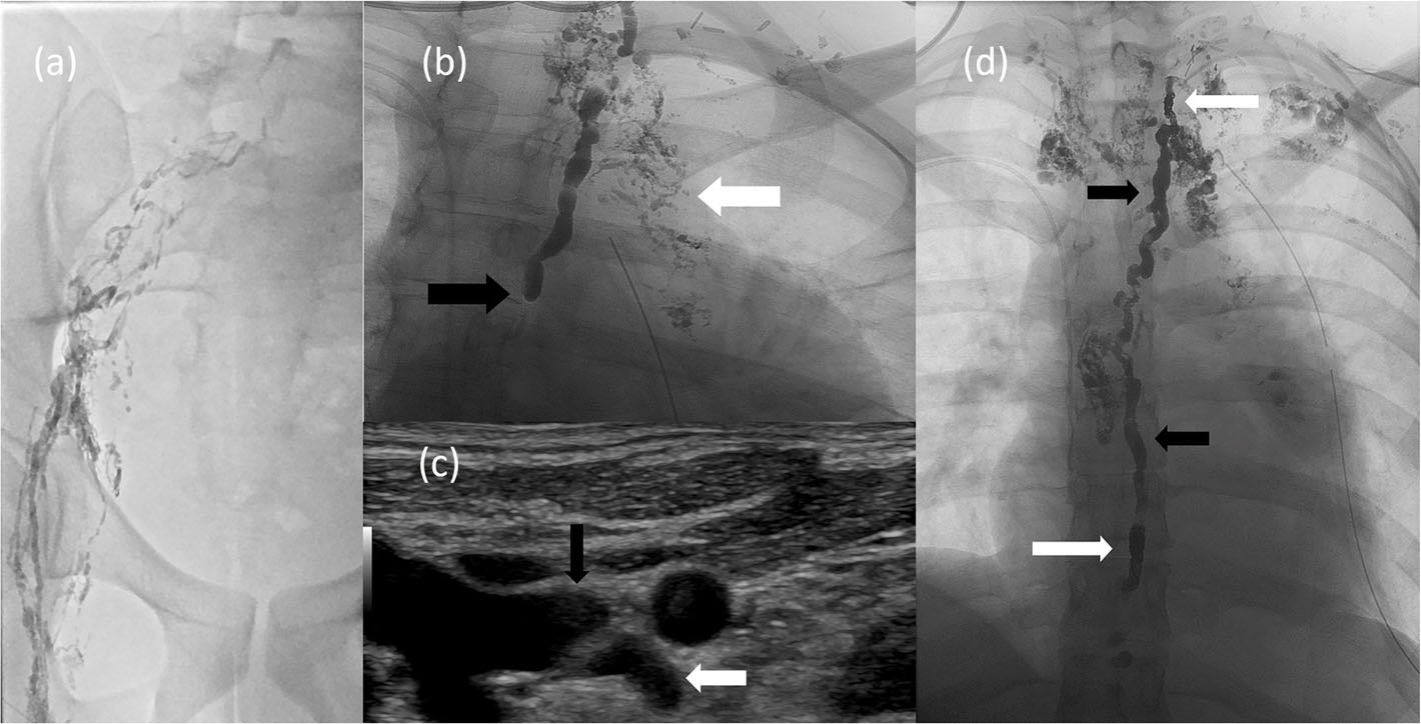
Intra-procedural findings. Lymphangiogram after ethiodol injection into a groin lymph node **(a)** revealed abnormal dilated and tortuous lymphatic channels in the right iliac chain. Fluoroscopic image **(b)** of the upper chest demonstrated a partially opacified thoracic duct terminating abruptly at the inferior margin (black arrow) and multiple abnormal, irregular lymphatic channels (white arrow) in the mediastinum corresponding to the CT image in Fig. 1. The abrupt termination was likely due to a valve. This was crossed without much difficulty with a microsystem. Grayscale ultrasound image **(c)** of the thoracic duct (white arrow) draining into the left subclavian vein (black arrow) at the venous angle. Intra-procedural fluoroscopic image **(d)** of the thoracic duct demonstrating embolization coils (white arrows) at the inferior and superior aspects of the duct. The intervening segment of thoracic duct was embolized with radiopaque NBCA (black arrows). A left chest tube and right PICC are present

Interventional radiology was consulted for evaluation of the chylothorax, which was presumed to be arising from the cut surface of the lymphatic-venous malformation. Lymphangiography was performed utilizing ethiodol injected from bilateral groin nodes. This revealed abnormal dilated and tortuous lymphatic channels in the right iliac chain (Fig. 2a), retroperitoneum, and mediastinum corresponding to the low-density structures identified on the prior CT exam. Ultrasound evaluation of the right iliac region demonstrated lesions consistent with microcystic (< 2 cm) lymphatic malformation. The abnormality involved the abdominal lymphatics. The thoracic duct was opacified but no discernible cisterna chyli was identified.

Attempts at accessing the lymphatic vessels in the region of the cisterna chyli with a 20 Gauge needle (Cook, Bloomington, IN) were successful but the wire could not be advanced to the thoracic duct (given lack of in-line lymphatic vessels from the upper abdomen to the thoracic duct). Hence, imaging of the chest was performed and a partly opacified thoracic duct was visualized (Fig. 2b). Ultrasound evaluation of the left supraclavicular region demonstrated a sonographically visible thoracic duct draining into the subclavian vein at the venous angle (Fig. 2c). The thoracic duct was catheterized from the left neck under ultrasound guidance using a 21 gauge needle through the subclavian approach. Over the 0.018 in. wire, we placed a transitional dilator. Two 0.018 in. wires were advanced through the 5 French catheter. Two microcatheters were then advanced into the thoracic duct. Coil embolization (Nester Coils, Cook Medical, Bloomington, IN) was first performed at the superior aspect of the thoracic duct close to the lymphovenous junction to prevent leakage of N-Butyl cyanoacrylate (NBCA) into the venous system. Coil embolization was performed at the inferior aspect of the thoracic duct through the more inferior of the microcatheters and then the thoracic duct and the lymphatic malformation/leakage at the level of the aortic arch were embolized with NBCA (ratio of ethiodol to NBCA was 4:1) (Fig. 2d). The benefit of the two microcatheters was the safety the second microcatheter provided. We were able to embolize the proximal and distal thoracic duct with coils and inject glue under some pressure. This proximal and distal coiling allowed the glue to percolate into the lymphatic malformation without leaking forward into the abdominal lymphatics (of no benefit) or back into the venous system (risk of glue embolization).

**Fig. 3:**
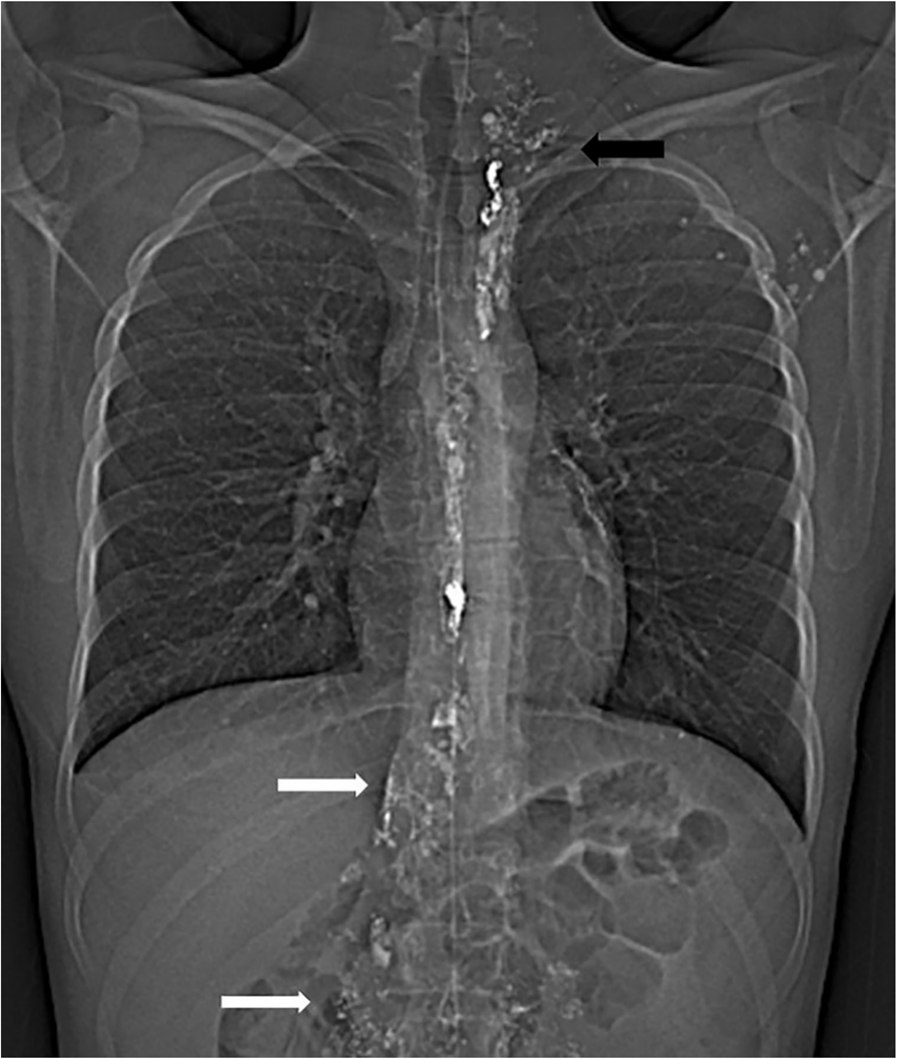
Post-procedural CT topogram demonstrating embolization coils and NBCA through the length of the thoracic duct. Ethiodol also opacifies portions of the malformation in the abdomen (white arrows) and mediastinum/left neck (black arrow) along with left axillary lymph nodes

Following the lymphangiogram and thoracic duct embolization procedure, the patient had a rapid recovery. Left chest tube output dropped dramatically and then tapered off completely over the following days. Seven days after the procedure, the chest tube was removed and the patient was discharged home. At clinic follow up 2 weeks later, he continued to do well with no re-accumulation of pleural fluid. A CT was performed with appropriate deposition of the embolic material in the lymphatic system in the chest (Fig. 3).

## Discussion

This case illustrates the challenges of accurately diagnosing and treating mediastinal and abdominal lymphatic malformations that may remain occult until adulthood. Appropriate imaging such as ultrasound and magnetic resonance imaging (MRI) are integral to the appropriate diagnosis of these pathologies. Complications of surgical excision include lymphatic leaks that may be refractory to conservative management. Further, in the presence of lymphatic malformations, the normal anatomy of lymphatic ducts is often altered precluding conventional thoracic duct embolization. Traditionally, thoracic duct embolization was performed through transabdominal approach, consisting of pedal lymphangiography, direct transabdominal catheterization of cisterna chyli and subsequently, antegrade catheterization of thoracic duct (Cope & Kaiser, 2002). However, this was technically challenging particularly in patients with obesity or deep inspiratory efforts, with a slight potential risk for injury to visceral organs (Guevara et al., 2016; Itkin & Chen, 2011) Over time, to overcome the challenges, different techniques such as retrograde approach have been developed where access is obtained in basilic, brachial, or even femoral vein and thoracic duct is catheterized via the lymphovenous junction (Kariya et al., 2018). In this case, we did not use retrograde thoracic duct access at the lymphovenous junction using venous access. This was due to the easy visualization of the thoracic duct draining into the vein on ultrasound which allowed for a straight trajectory allowing us to bypass the angles/valves at the lymphovenous junction. In this case, a method of accessing the terminal aspect of the thoracic duct through the subclavian vein was utilized due to the abnormal anatomy of the abdominal lymphatics (Kariya et al., 2018). The thoracic duct can be visualized sonographically as a tubular structure in the left neck at the junction of the subclavian and internal jugular veins. Catheterization of the duct in this location is challenging but can be performed if adequately visualized on ultrasound or fluoroscopy.

## Conclusion

In patients with altered lymphatic anatomy, conventional thoracic duct embolization might not be feasible. In these patients, using subclavian approach is a promising alternative to perform the procedure.

## Data Availability

Not Applicable.

## Abbreviations

CMV: Cytomegalovirus
LDH: Lactate dehydrogenase
PET: Positron emission tomography
CT: Computed tomography
VATS: Video-assisted thoracoscopic surgery
NBCA: N-Butyl cyanoacrylate
MRI: Magnetic resonance imaging
